# Complete Remission Obtained with Azacitidine in a Patient with Concomitant Therapy Related Myeloid Neoplasm and Pulmonary Mucormycosis

**DOI:** 10.4084/MJHID.2013.048

**Published:** 2013-07-10

**Authors:** S. Capria, F. De Angelis, G. Gentile, S.M. Trisolini, S. Brocchieri, M. Canichella, P. Chiusolo, A. Micozzi, R. Foà, G. Meloni

**Affiliations:** 1 Department of Cellular Biotechnologies and Hematology, “Sapienza” University of Rome, Rome, Italy; 2 Department of Hematology, Catholic University of the Sacred Heart, Rome, Italy; 3 Department of Radiologic Sciences, Azienda Policlinico Umberto I, “Sapienza” University of Rome, Rome, Italy

## Abstract

Mucormycosis is the third cause of invasive mycosis after candidiasis and aspergillosis in AML patients, representing a poor prognostic factor associated with a high rate of fatal outcome. We report a case of a patient with AML and a concomitant pulmonary mucormycosis at diagnosis, who obtained a complete remission both of her AML and of the fungal infection. The incidence of the infection at the onset of leukemia is extremely unusual, and, to our knowledge, the sporadic cases reported in the literature are included in heterogeneous series retrospectively examined.

In our case, Liposomal Amphotericin B as single agent appeared incapable of controlling the infection, so anti-infective therapy was intensified with posaconazole and simultaneously antileukemic treatment with 5-azacitidine was started, with the understanding that the only antifungal treatment would not have been able to keep the infection under control for a long time if not associated with a reversal of neutropenia related to the disease.

We observed a progressive improvement of the general conditions, a healing of pneumonia and a complete remission of the leukemic disease, suggesting that a careful utilization of the new compounds available today, in terms of both antifungal and antileukemic treatment, may offer a curative chance a patient who would have otherwise been considered unfit for a potentially curative therapeutic strategy.

## Introduction

Despite the improvements attained over the years, acute myeloid leukemia (AML) remains a life-threatening malignancy. The chance of cure depends upon the possibility of achieving a complete remission, which is obtained in 50–80% of adult patients treated with intensive chemotherapy. Patients enrolled in standard chemotherapy protocols have first to be considered eligible for intensive therapy on the basis of standard inclusion criteria. The presence of an active uncontrolled infection at baseline represents one of the most severe factors that negatively impacts on treatment-related mortality (TRM), independently of the co-morbidity index scoring. For this reason, patients who present with a severe infection at the time of the diagnosis of AML are generally considered unfit for the commonly used induction treatment strategies. Mucormycosis is an emerging opportunistic infection, sustained by ubiquitous filamentous fungi of the Zygomicetes order, with different patterns of clinical manifestations: rhino-cerebral, pulmonary, cutaneous, gastrointestinal, and disseminated forms, although uncommon localizations are also described.^1^ Major risks for developing Mucormycosis are represented by concomitant diseases associated with persistent immunosuppression, such as uncontrolled diabetes mellitus, trauma, solid organ or bone marrow transplantation, burns and hematological malignancies. In AML patients, it is the third cause of invasive mycosis after candidiasis and aspergillosis, it is generally observed during the aplastic phase following intensive chemotherapy treatment or allogeneic stem cell transplantation and still remains associate with a mortality rate of 50–80%.^2^,^3^ In view of its highly aggressive behavior, it should be treated with appropriate antifungal therapy immediately after the diagnosis; indeed, a six days’ delay in starting the appropriate therapy is associated with a doubled mortality rate.^4^ Generally, the presence of active mucormycosis contraindicates aggressive antineoplastic treatments.

## Case Report

We report herein a case of a patient with AML and a concomitant pulmonary mucormycosis at diagnosis, who obtained a complete remission both of her AML and of the fungal infection. The incidence of the infection at the onset of leukemia is extremely unusual, and, to our knowledge, the sporadic cases reported in the literature are included in heterogeneous series retrospectively examined.^5^ Probably in our patient a potential role of other underlying predisposing conditions can be hypothesized, like previous chest radiotherapy for breast cancer.

**Figure 1:**
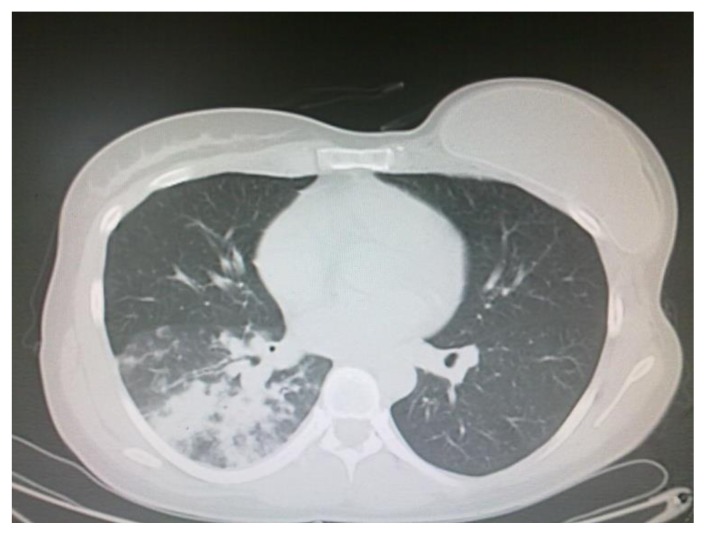
CT Scan at diagnosis of AML: bilateral basal parenchymal consolidation with a consensual right pleural effusion.

In November 2011, a 35-year-old woman presented to the Emergency Department with fever, fatigue and worsening dyspnea. At physical examination, bilateral later cervical and suvraclavear lymphadenopathies were found, while spleen and liver were not palpable. The full blood cell count showed: hemoglobin concentration 10.4 gr/dl, white cell count 1.4 × 10^9^/L, neutrophil count 0.4 × 10^9^/L, platelet count 181 × 10^9^/L. On the basis of these findings, patient was referred to the hematology unit for further investigations. The clinical history documented a breast ductal adenocarcinoma treated five years earlier with radical mastectomy followed by adjuvant chemotherapy with docetaxel, capecitabine, trastuzumab and anterior chest wall radiotherapy. The bone marrow aspirate was markedly hypocellular with rare myeloperoxidase (MPO) negative blasts and basophilic cytosol; rare granulations were observed. Flow cytometry immunophenotyping, performed on the hypocellular sample, documented the presence of 21% CD34/CD13/CD33/HLADR positive and MPO/CD41/CD61/CD117/CD7/CD56 negative myeloid blasts. The bone marrow biopsy showed: “Grade III of fibrosis, with bone marrow sites fully occupied (90%) by CD34 positive, Terminal Desoxynucleotidil Transferase +/−, MPO negative blasts”. These features where consistent with the diagnosis of AML. Cytogenetic analysis resulted in a normal karyotype (46 XX); RT-PCR was negative for molecular abnormalities commonly associated with AML The chest CT scan performed at the time of hospital admission revealed the presence of a bilateral basal parenchymal consolidation (45 × 47 mm in the basal right lobe, 21 ×28 mm in the left basal lobe) with a consensual right pleural effusion (Figure 1); no involvement of paranasal sinuses was reported. Based on the CT scan results and on the presence of fever, a broad spectrum antibiotic therapy was instituted on the same day, first with piperacillin/tazobactam for 6 days without response, followed by meropenem, levofloxacin and linezolid due to the worsening of the lung involvement at chest CT scan; blood cultures were persistently negative. A diagnostic work-up for fungal infections showed negativity of galactomannan and plugs, but allowed the detection of Mucor species in the sputum.

**Figure 2:**
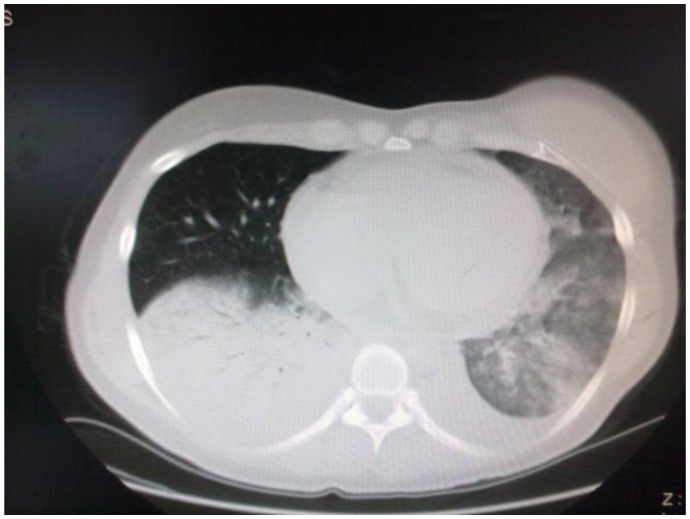
CT Scan before posaconazole treatment.

**Figure 3:**
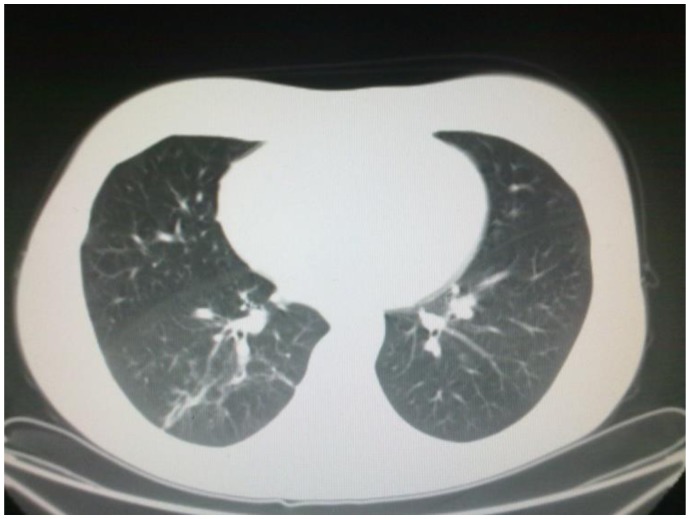
Resolution of the infection.

Liposomal amphotericin B (L-AmB) at 5 mg/kg/day was then started after 9 days of antibiotic therapy. The clinical conditions worsened, with recurrent episodes of hemoptysis, persistent positivity for mucor in expectorations and parenchymal consolidation of pulmonary lesions, with a further enlargement of the bilateral nodular lesions (71 × 64 mm in the basal right lobe, 46 × 30 mm in the basal left lobe) and presence of pleural effusion (Figure 2); on the basis of this life threatening condition, we attempted to improve antifungal therapy with the addition of posaconazole (600 mg/day) and, at the same time, to start chemotherapy with azacitidine 75 mg/m^2^ for 7 days. The choice of intensifying anti-infective therapy and simultaneously start the antileukemic treatment was guided by the knowledge that the only antifungal treatment would not have been able to keep the infection under control for a long time if not associated with a reversal of neutropenia related to the disease. Within two days, the patient became afebrile with an improvement of the clinical status; a CT scan performed 14 days later showed a reduction of the lung lesion. The patient received three cycles of azacitidine at three weeks interval. At the end of the third cycle, a restaging of the hematological disease with a bone marrow biopsy was carried out and showed a picture of complete hematologic remission; the patient lacked a matched related sibling and was scheduled for a matched unrelated donor (MUD) allogeneic stem cell transplant. While waiting for the transplant, she underwent six further cycles of azaticidine which allowed maintaining the complete remission. Chest CT scans showed a progressive resolution of the pulmonary lesions (Figure 3); L-AmB was stopped after three months of treatment, while posaconazole was continued for further five months, until the patient received the transplant.

**Figure 4:**
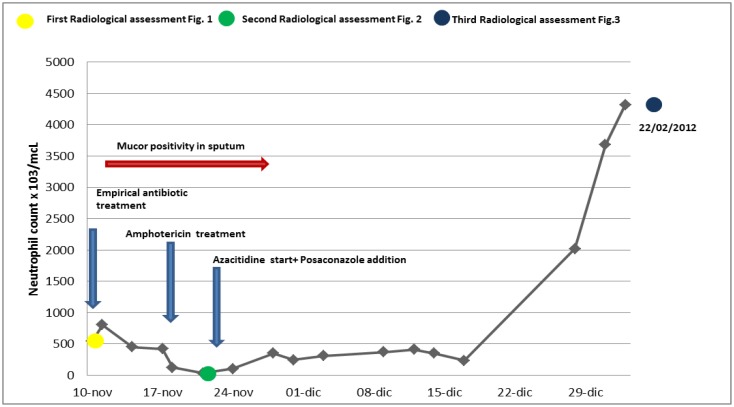
Relationship between neutropenia and antibiotic/antifungal therapies during induction phase.

Figure 4 shows the relationship between neutropenia and antibiotic/antifungal therapies during induction phase.

In June 2012, the MUD transplant was performed; because of an engraftment failure, the patient underwent a second stem cell infusion from the same donor in August 2012, which was followed by a satisfactory stem cell engraftment. Graft-versus-host disease (GVHD) prophylaxis included methotrexate and cyclosporine. During this period, posaconazole prophylaxis was continued and no evidence of Mucor reactivation was documented. Despite an appropriate immunosuppressive therapy, the patient experienced a severe hepatic, gastrointestinal, cutaneous acute GVHD; rituximab and photoapheresis procedures were promptly started but the patient died at day +170 due to persistent severe GVHD.

During the last years, adjuvant chemotherapy and radiotherapy for breast cancer have significantly improved the number of long-term survivors, but these patients are at high risk of developing a therapy-related myelodysplastic syndrome and/or an AML (t-MDS/AML). Despite recent progress in AML treatment, t-AML is still associated to a poor prognosis, with a lower complete remission rate following conventional induction treatment and a shorter median survival compared to de novo AML.^6^ Patients with t-AML are therefore candidate to an allogeneic transplant if the performance status, donor availability and former neoplastic disease allow this approach.^7^ Azacitidine, a nucleoside analog of cytidine with hypometilating effects, is approved for the treatment of high-risk MDS and AML with 20–30% bone marrow blast count, and has shown a significant survival benefit compared to conventional chemotherapy regimens. The main advantage of the use of azacitidine consists in the possibility of obtaining a complete remission of disease without passing through a phase of deep aplasia. For this reason its validity has also been demonstrated in patients unfit for more intensive therapies.^8^ Although Azacitidine is not indicated in patients with high blast count AML, its use as first line treatment in our patient was justified by the presence of a life-threatening infection, which hampered the use of more aggressive therapies. Mucor infection, as previously specified, represents a poor prognostic factor associated with a high rate of fatal outcome. To our knowledge, no reports are present in the literature regarding the association of AML with a concomitant Mucormycosis infection at diagnosis. In a large cohort study performed in Italy on more than 11.000 hematological patients, authors reported an invasive fungal infection (IFI) frequency of 4.6% with mucormicosis at 0.1%; in this study, the IFI-attributable mortality rate specific for mucormicosis (64%) was the highest among fungal infections.^9^ Lung seems to be involved more frequently than other anatomic sites,^10^ in line with the clinical presentation in our patient. In this case, L-AmB as single agent appeared incapable of controlling the infection, as did instead the association with posaconazole. Although more studies are needed to confirm the efficacy of an association therapy with L-AmB and posaconazole,^11^ our experience demonstrates that, despite the presence of a potentially fatal fungal infection at the time of presentation, a tailored therapy for AML is feasible. A careful utilization of the new compounds available today, in terms of both antifungal and antileukemic treatment, may offer a chance to undergo an allografting procedure in a patient who would have otherwise been considered unfit for a potentially curative therapeutic strategy.
